# Supplementary material for: Alterations of the gut microbiota in type 2 diabetics with or without subclinical hypothyroidism
Source: PeerJ. 2023 Apr 13;11:e15193. doi: 10.7717/peerj.15193 (PMC10106085; doi:10.7717/peerj.15193)
Supplement: Supplemental Information 1 — Data are shown as mean±SD. FPG,fastingplasma glucose; PBG, postprandial blood glucose; HbA1c, hemoglobin A1c; TSH, thyroid stimulating hormone; FT3, free triiodothyronine; FT4, free thyroxine; TCH, total cholesterol; TG, triglyceride; HDL-C, high density lipoprotein cholesterol ; LDL-C, low density lipoprotein cholesterol; TBIL, total bilirubin; DBIL, direct bilirubin; IBIL, indirectbilirubin; ALB, albumin. [file peerj-11-15193-s001.docx]

| **Supplemental Table S1.** **Clinical characteristics of T2D group and T2D_SCH group** | | | |
| --- | --- | --- | --- |
| Parameters | T2D | T2D_SCH | *P* value |
| FPG (mmol/L) | 10.16±3.21 | 12.33±4.56 | 0.071 |
| PBG (mmol/L) | 20.27±6.08 | 21.72±5.80 | 0.449 |
| HbA1c (%) | 9.15±2.28 | 9.51±2.24 | 0.622 |
| TSH (µIU/ml) | 2.05±1.02 | 7.99±6.35 | ＜0.001 |
| FT3 (pmol/L) | 4.64±0.46 | 4.67±0.51 | 0.810 |
| FT4 (pmol/L) | 16.95±2.10 | 15.37±2.24 | 0.025 |
| TCH (mmol/L) | 4.94±1.00 | 4.71±1.29 | 0.520 |
| TG (mmol/L) | 2.33±1.68 | 2.81±1.10 | 0.321 |
| HDL-C (mmol/L) | 1.26±0.30 | 1.10±0.22 | 0.074 |
| LDL-C (mmol/L) | 2.73±0.72 | 2.49±0.97 | 0.346 |
| VitD3 (ng/ml) | 20.51±9.60 | 17.24±5.39 | 0.228 |
| TBIL (μmol/L) | 16.84±6.99 | 15.21±7.39 | 0.472 |
| DBIL (μmol/L) | 3.59±1.20 | 2.86±0.99 | 0.061 |
| IBIL (μmol/L) | 13.35±6.17 | 12.35±6.57 | 0.654 |
| ALB (g/L) | 41.66±3.25 | 42.33±3.07 | 0.510 |
| Course of T2D (year) | 5.72±6.00 | 7.20±6.16 | 0.413 |
| Use of insulin (Yes; %) | 63.33 | 66.67 | 0.826 |
| Use of metformin (Yes; %) | 83.33 | 80.00 | 0.542 |
| Data are shown as mean±SD.  FPG, fasting plasma glucose; PBG, postprandial blood glucose; HbA1c, hemoglobin A1c; TSH, thyroid stimulating hormone; FT3, free triiodothyronine; FT4, free thyroxine; TCH, total cholesterol; TG, triglyceride; HDL-C, high density lipoprotein cholesterol; LDL-C, low density lipoprotein cholesterol; TBIL, total bilirubin; DBIL, direct bilirubin; IBIL,  indirect bilirubin; ALB, albumin. | | | |

**Supplemental Table S2. Spearman's rank correlation analysis of AMY1 CN and gut microbes**

| Species classification | r | *P* Value |
| --- | --- | --- |
| **Phylum** |  |  |
| Proteobacteria | 0.380 | 0.010 |
| Bacteroidetes | -0.318 | 0.034 |
| **Genus** |  |  |
| *Granulicatella* | 0.339 | 0.026 |
| *Klebsiella* | 0.314 | 0.041 |
| *Escherichia/Shigella* | 0.447 | 0.003 |
| *Rothia* | 0.367 | 0.016 |
| *Sporobacter* | -0.376 | 0.013 |
| *Anaerovorax* | -0.350 | 0.021 |
| *Clostridium_IV* | -0.385 | 0.011 |
| *Alistipes* | -0.391 | 0.010 |
| *Bacteroides* | -0.368 | 0.015 |
| *Parabacteroides* | -0.329 | 0.031 |
