# Supplementary figures and images for: Alterations of the gut microbiota in type 2 diabetics with or without subclinical hypothyroidism
Source: PeerJ. 2023 Apr 13;11:e15193. doi: 10.7717/peerj.15193 (PMC10106085; doi:10.7717/peerj.15193)

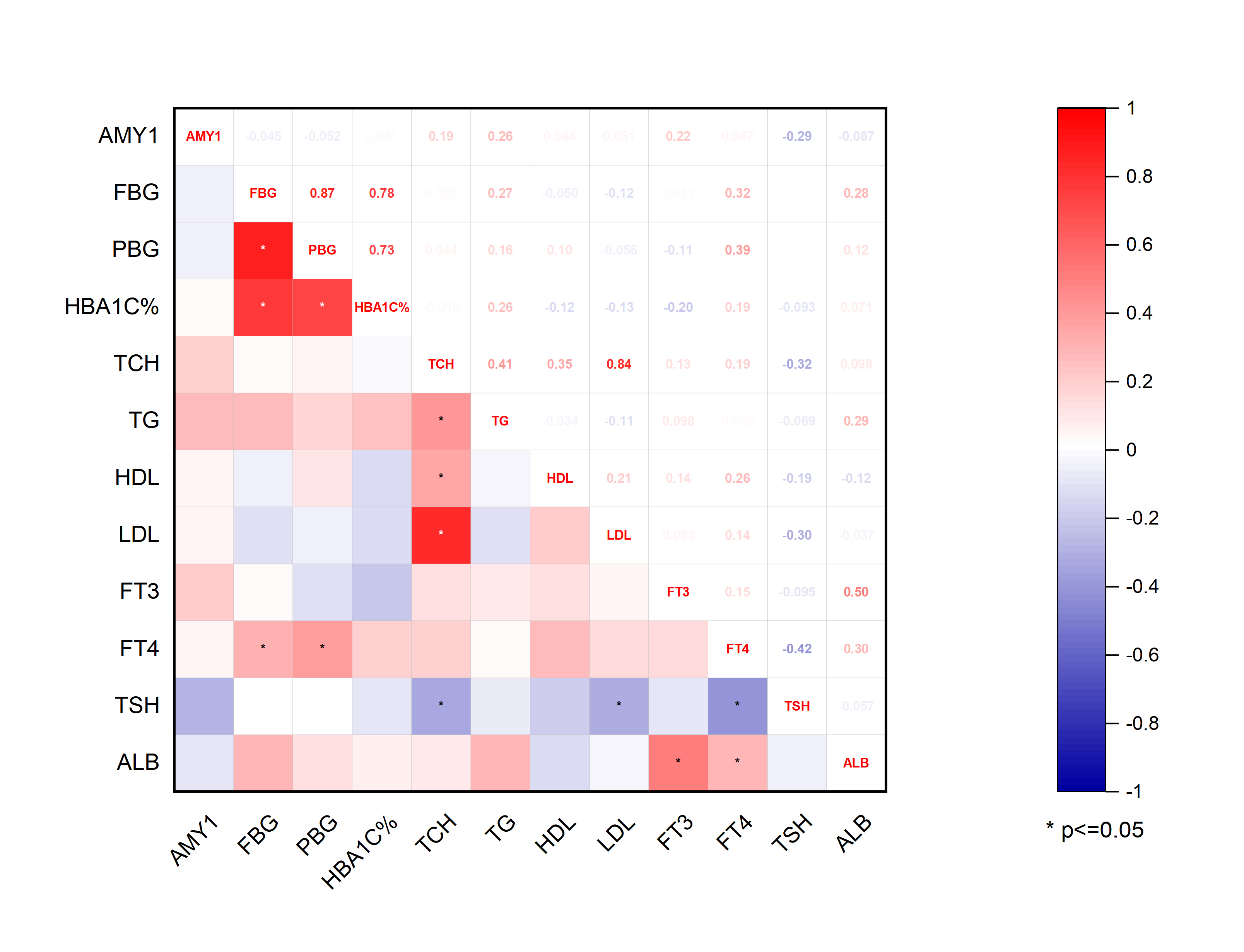

Supplement: Supplemental Information 2 — The asterisk indicates P ≤ 0.05. And the number indicates the value of Pearson’s correlation coefficient. Red represents positive correlation and blue represents negative correlation. [file peerj-11-15193-s002.jpg]

A

PCoA

[PERMANOVA] F-value: 1.1388; R-squared: 0.025801; p-value &lt; 0.276

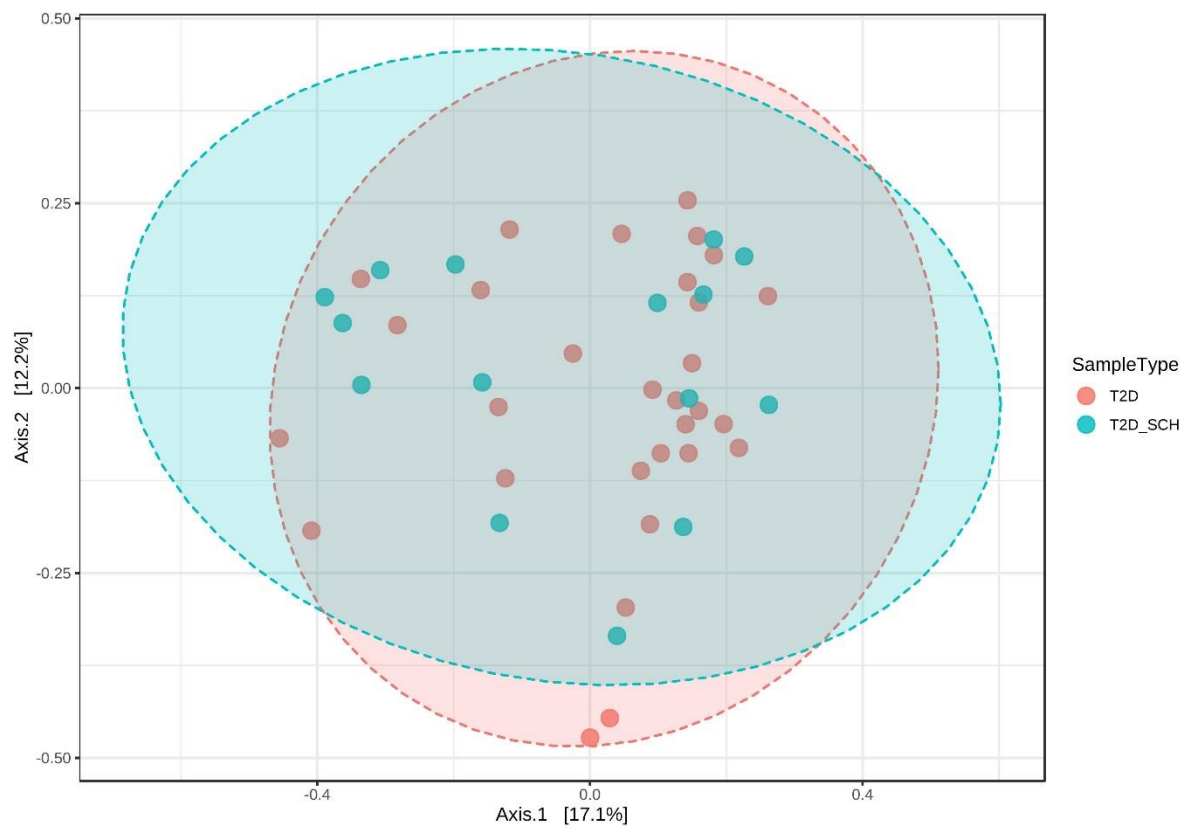

B

PLS\_DA

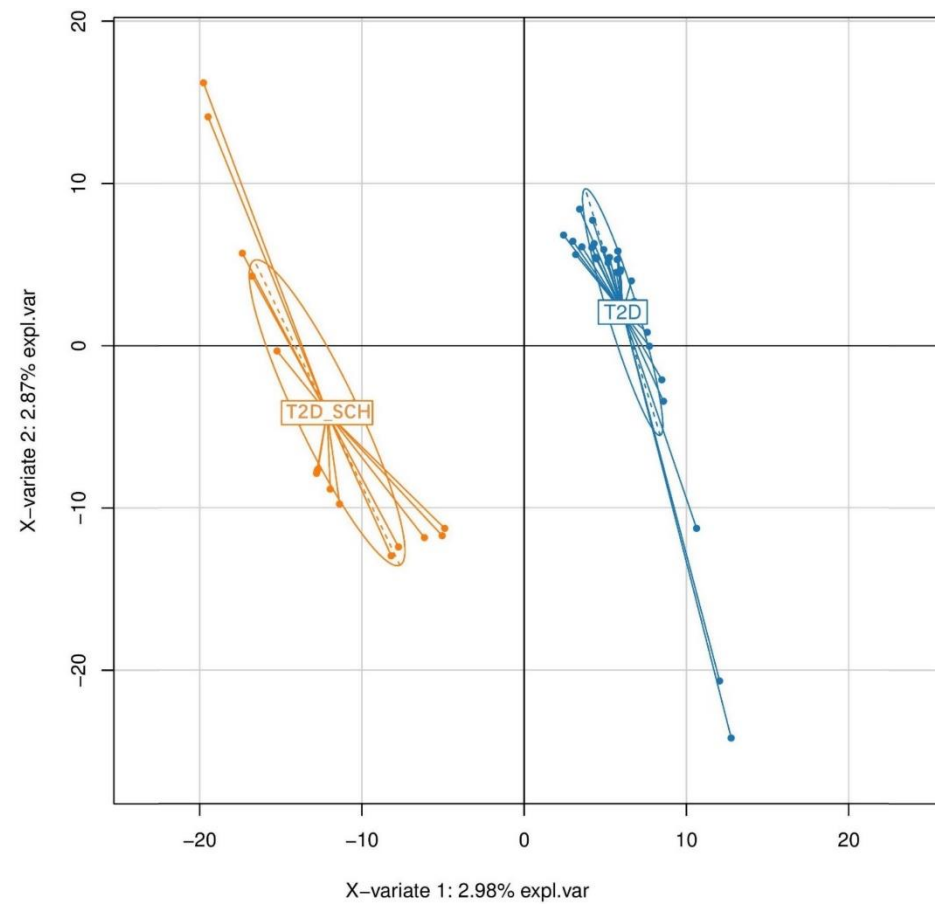

Supplement: Supplemental Information 3 [file peerj-11-15193-s003.pdf]

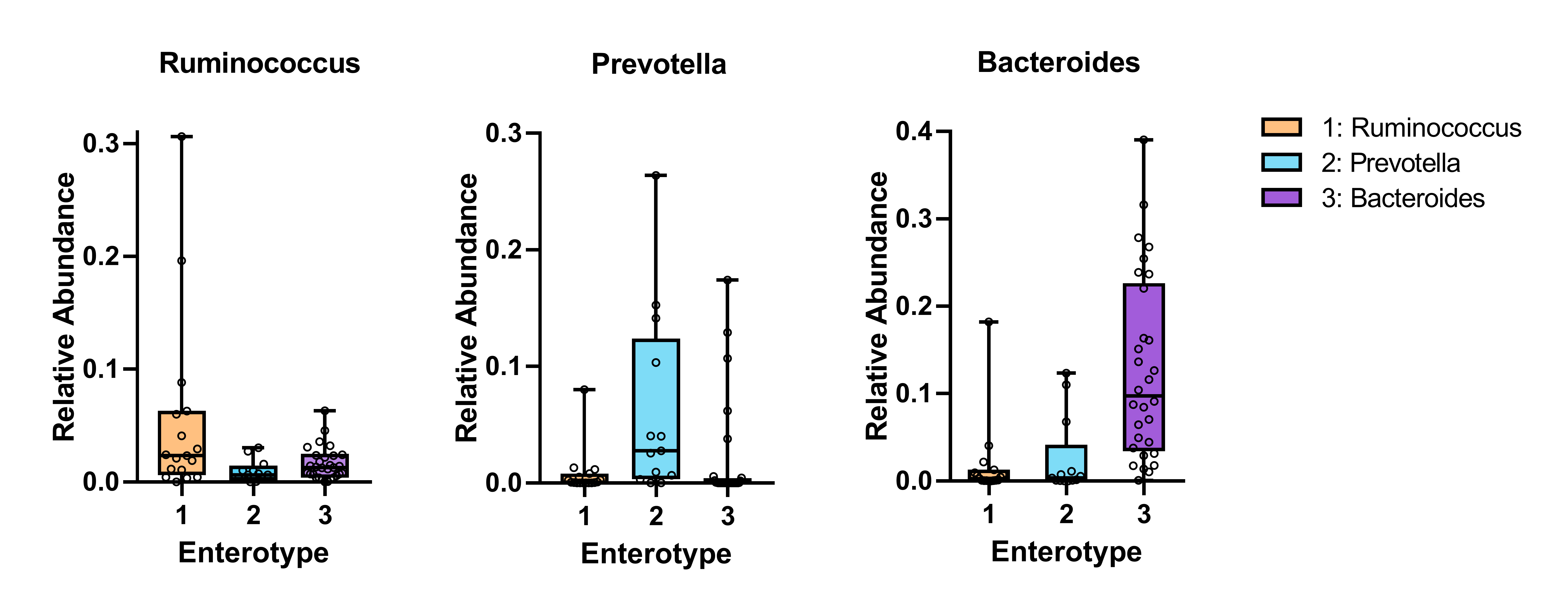

Supplement: Supplemental Information 4 [file peerj-11-15193-s004.png]
